# “Boosting” Surveillance for a More Impactful Public Health Response During Protracted and Evolving Infectious Disease Threats: Insights From the COVID-19 Pandemic

**DOI:** 10.1089/hs.2023.0046

**Published:** 2023-09-27

**Authors:** Saba A. Qasmieh, McKaylee M. Robertson, Denis Nash

**Affiliations:** Saba A. Qasmieh, MPH, is a Research Scientist, Institute for Implementation Science in Population Health, and a PhD Student, Department of Epidemiology and Biostatistics, Graduate School of Public Health and Health Policy, University of New York, New York, NY.; McKaylee M. Robertson, PhD, MPH, is an Investigator, Institute for Implementation Science in Population Health, University of New York, New York, NY.; Denis Nash, PhD, MPH, is Executive Director, Institute for Implementation Science in Population Health, and Distinguished Professor of Epidemiology, Department of Epidemiology and Biostatistics, Graduate School of Public Health and Health Policy, University of New York, New York, NY.

**Keywords:** COVID-19, Surveillance, Bias, Population-based surveys, Infectious disease, Disease burden

## Introduction

*Good surveillance does not necessarily ensure the making of the right decisions, but it reduces the chances of [making the] wrong ones.* – Alex Langmuir, 1963

Emerging or reemerging infections, as well as other pathogens of public health significance, require robust surveillance systems. These surveillance systems should accurately assess the health threat and have goals that are aligned with what is often a multisectoral public health response (ie, containment, control, mitigation, elimination).^1^ In the context of a protracted public health emergency like the COVID-19 pandemic, surveillance systems must also adapt to inform public health interventions. The goals of the public health response to the COVID-19 pandemic have been dynamic and manifold, including preventing severe disease, death, and infection sequelae (and related inequities); detecting and minimizing the impact and disruption from surges; maximizing knowledge, access, and uptake of testing and key pharmaceutical interventions (vaccines, boosters, and antivirals); and informed and timely health education and risk communication to the public on disease risk and personal protective behaviors.

As the COVID-19 pandemic enters its fourth year, it continues to pose a major public health threat, with excess deaths,^2,3^ decreases in US life expectancy, and increases in the burden of long COVID.^4^ Yet the utility of routine COVID-19 surveillance in the United States has become increasingly limited,^5^ partly because the surveillance systems do not collect health indicators that are aligned with public health goals, which themselves are not always clearly stated. In addition, a dynamic and poorly characterized backdrop of underlying hybrid immunity has further complicated efforts to monitor transmission and disease severity.^6^ The COVID-19 pandemic response has focused primarily on increasing uptake of vaccines and antiviral treatments, largely in an untargeted way.

The goal of this commentary is to provide a case for boosting the existing COVID-19 public health surveillance strategy to one that is more aligned with public health goals of mitigation and control, including more rapid monitoring of acute and postacute SARS-CoV-2 infection burden and related inequities. We draw on the experience of the COVID-19 pandemic in the United States to identify important gaps in the surveillance of protracted health emergencies that require rapid and reliable monitoring and active dissemination of dynamic health indicators on key sociodemographic, behavioral, and immunological dimensions. We highlight the use of routinely and strategically deployed population-representative surveys as a possible remedy to address key surveillance gaps. We draw on insights gained from 3 strategically deployed population-based surveys in New York City and the United States during major Omicron variant surges, including BA.1,^7^ BA.2,^8^ and BA.4/BA.5.^9,10^

## Surveillance Gaps in Monitoring Transmission and Severity

Standard COVID-19 surveillance in the United States employs multiple approaches. A surveillance approach, based on case numbers and testing, informs disease burden estimates and potential intensity of transmission and enables monitoring for genomic characteristics of new variants.^11^ A syndromic surveillance approach is used for early detection of suspected COVID-19 cases that present to emergency departments.^12^ Hospital-based sentinel surveillance,^13^ monitoring of hospitalization rates, and COVID-19-specific deaths allow for assessing the severity of disease. Additionally, monitoring staffed inpatient beds occupied by COVID-19 patients reflects local healthcare system usage and remaining capacity that aims to monitor transmission that leads to severe disease.^11^

Key gaps in COVID-19 surveillance include (1) a lack of or incomplete information on sociodemographic and timely information on epidemiologic risk factors driving morbidity and mortality, including vaccination status; (2) incompleteness due to underreporting and exclusion of at-home tests; and (3) inadequate monitoring of long COVID outcomes. Each of these contributes to an inability to accurately monitor and disseminate information on population-level morbidity and mortality as well as health inequity-related metrics. These gaps could result in a poor understanding of the population health risk posed by COVID-19, potentially leading to suboptimal, ineffective, or poorly targeted public health response to surges.

Case and testing-based indicators are biased metrics of COVID-19 burden in the population,^14^ as they reflect an incomplete subset of infections in the population (patients who present for testing or care), and do not consistently capture socioeconomic or adequately detailed demographic factors that may drive healthcare-seeking behavior.^15^ Case-and testing-based surveillance also does not capture individuals who test positive at home without a confirmatory diagnostic test with a provider,^16^ while the proportion who test exclusively at home is likely increasing over time.^17^ Furthermore, the completeness of reporting on cases and tests by healthcare, laboratory, or other providers in the community has not been systematically evaluated in the United States and may be negatively influenced by surges in transmission, test availability, testing demand, or a combination^18^ (Figure).

In light of these limitations, the US Centers for Disease Control and Prevention (CDC) shifted toward the use of hospitalization rates and intensive care unit bed capacity to determine COVID-19 community transmission levels and inform county-level mitigation measures.^5^ While this approach addresses a major priority (prevention of severe disease and death), hospital-based data are not timely indicators of transmission, and are therefore inadequate for informing strategies that prevent severe outcomes.^19^ Moreover, hospitalization data increasingly reflect a lack of access to care and a lack of uptake of boosters and antivirals among those at risk, making hospitalization data biased indicators of community transmission. Wastewater surveillance for SARS-CoV-2 has demonstrated a potential critical role in the timely detection of surges,^20^ but wastewater metrics are challenging to standardize and gaps remain in contextualizing wastewater indicators with epidemiological data for policy decisions.^21^ Given these gaps, the COVID-19 surveillance approach in the United States has consistently underdelivered in the goal of providing timely and useful health indicators to inform a more effective public health response^22^ (Table).

## Survey-Based Approaches to Improve Public Health Surveillance

Probability-based population-representative surveys that are conducted routinely and at strategic time points (eg, during surges), with survey findings shared promptly, can be a feasible surveillance tool for assessing the true burden of COVID-19 cases and related outcomes to inform the public health response.^23,24^ As of July 2023, the World Health Organization (WHO) guidance on public health surveillance for COVID-19 continues to recommend enhanced surveillance approaches and special studies to describe and monitor groups at highest risk for exposure or severe disease, characterize severity, assess long COVID burden and risk factors, and estimate vaccine effectiveness and level of population immunity.^25^ Special studies in the United States have been conducted to elucidate disparities in SARS-CoV-2 infections and hospitalization,^26,27^ severity of SARS-CoV-2 variants,^28^ vaccine effectiveness and the need for boosters,^29^ and underlying immunity due to vaccination and prior infection.^30^ However, typical approaches to gathering this information are retrospective, not timely, and prone to limitations given that they often utilize (biased) case and testing indicators from passive surveillance.

**Figure.**
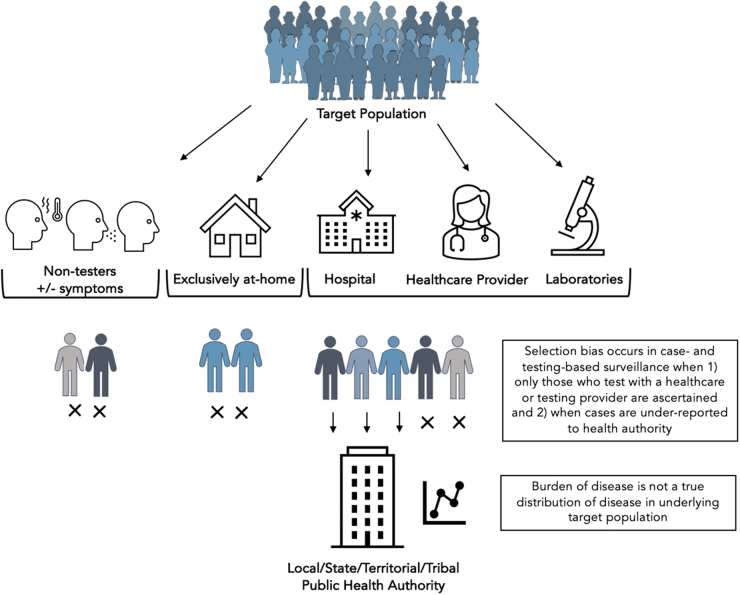
Selection bias in case- and testing-based surveillance when estimating the burden of SARS-CoV-2 infections. An X under a person in the figure indicates that their infections are not captured in case-/testing-based surveillance.

The use of population-representative surveys as a surveillance tool has been demonstrated in the United Kingdom.^31,32^ These surveys collect self-reported data combined with biomarker information on SARS-CoV-2 infection to generate valid estimates of prevalence^33^ and detect population-level immunity due to acquired infections and/or vaccination.^34^ By applying a consistent survey sampling methodology, it is possible to ascertain types of recently taken SARS-CoV-2 diagnostic tests by self-report, along with their results, which could provide less resource-intensive estimates of prevalence that circumvent biases arising from convenience sampling in passive surveillance.^35,36^ Additionally, for those with symptoms who do not test, collecting survey data on their symptoms and recent epidemiologic linkages with confirmed or probable close contacts allows for the measuring of a portion of undiagnosed cases. Alongside tests and test results, surveys can also capture indicators of COVID-19-related risk factors and behaviors, vaccination status and related barriers, prior infection(s), and antiviral awareness/uptake that are essential for understanding the distribution of infection and severe disease risk across key sociodemographic, immunological, and behavioral factors.

Despite the widespread use of rapid assessments via telephone or mobile-based applications to collect information on COVID-19 knowledge,^37^ attitudes,^38^ perceptions,^39^ and vaccine hesitancy^40,41^ among behavioral risk factors during the pandemic,^42^ routine surveys have not been widely used to rapidly monitor the infection burden in communities and disseminate findings for decisionmaking. In 2022, our team deployed population-representative surveys out of concern for bias and missing information on key subgroups in routine case- and testing-based SARS-CoV-2 surveillance. Population-representative surveys in the United Kingdom informed our approach to generate less biased and more complete estimates of SARS-CoV-2 burden during surges when testing availability and access may have been limited. Further, given the increasing availability of at-home rapid tests at the time, and the lack of a mechanism to systematically capture information on home test results, we used this approach to directly ascertain known infection status based on at-home rapid tests among a representative sample of the population. We deployed 3 population-representative surveys of a random sample of national and New York City residents to estimate the burden of SARS-CoV-2 infections during surges of Omicron subvariants BA.1, BA.2, and BA.4/5.^7-10^ The surveys also asked about awareness and uptake of antiviral treatments as well as postacute sequelae (long COVID) of recent SARS-CoV-2 infection.

**Table:** Table. Attributes, Strengths, and Weaknesses of Different Surveillance Approaches for Capturing Key Information Needed to Inform the Public Health Response to the COVID-19 Pandemic

	Syndromic Surveillance^a^	Case- and Testing-Based Surveillance^b^	Surveillance Based on Hospitalization Data^c^	Surveillance Based on Number of Deaths^d^	Commercial Laboratory-Based Sentinel Surveillance (Remnant Sera)^e^	Wastewater Surveillance^f^	Survey-Based Surveillance^g^	Survey-Based Surveillance + SARS-CoV-2 Biomarker^h^
Spectrum of illness	Severe only	Broad	Severe only	Severe only	Broad	Broad	Broad	Broad
Timeliness (for surge detection)	Excellent	Very good	Fair	Fair	Poor	Excellent	Very good	Very good
Completeness of reporting	Excellent	unknown	Excellent	Excellent	Good	n/a	n/a	n/a
Representativeness^i^	Excellent	Fair	Excellent	Very good	Fair	Excellent	Very good	Very good
Sociodemographic detail		Fair	Fair	Good	Fair	–	Excellent	Excellent
Geographic specificity	Fair	Excellent	Excellent	Excellent	Excellent	Good	Excellent	Excellent
Knowledge/behaviors	–	–	–	–	–	–	Excellent	Excellent
Misinformation/disinformation spread	–	–	–	–	–	–	Excellent	Excellent
Captures asymptomatic infections	–	–	–	–	Excellent	Excellent	–	Excellent
Captures undiagnosed infections	–	–	–	–	Excellent	Excellent	Good	Good
Captures home-based testing	–	–	–	–	–	–	Excellent	Excellent
Vaccine hesitancy	–	–	–	–	–	–	Excellent	Excellent
Vaccination status	–	Fair	Fair	Fair	Excellent	–	Excellent	Excellent
Prior infection	–	–	–	–	Excellent	–	Very good	Very good
Comorbidities	–	Poor	Fair	Fair	–	–	Very good	Very good
Antiviral use	–	–	–	–	–	–	Excellent	Excellent
Long-term sequalae	–	–	–	–	–	–	Excellent	Excellent

^a^ Captures emergency department visits and hospitalizations; no diagnostic information. ^b^Limited to those testing positive with a provider/laboratory AND reported to health department. ^c^Has been criticized for challenges in distinguishing hospitalization for COVID-19 vs hospitalization with COVID-19 for capturing severity; surveillance based on hospitalization data also allows for the assessment of hospital bed capacity and intensive care unit bed capacity. ^d^Likely undercounts COVID-19 deaths. ^e^Prone to bias as it is limited to those who access the healthcare system and receive a blood draw; can capture prevalence of antibodies induced by both prior infection and vaccination.

^f^ Not calibrated to cases; sensitivity may change with high levels of population immunity. ^g^Prone to bias at response stage of survey; may require advanced statistical methods to ensure representativeness and validity (eg, weighting). ^h^Prone to bias at response stage of survey; may require advanced statistical methods to ensure representativeness and validity (eg, weighting); more expensive and less timely than without biomarkers. ^i^Representativeness refers to part of the spectrum of infection that each surveillance modality is targeting.

## Insights From Rapid Population-Representative Surveys of SARS-CoV-2 Prevalence

### Estimating True Burden of Infection in Relation to Number of Reported Cases

A primary strength of population-representative surveys that estimate prevalence is their ability to assess the extent to which SARS-CoV-2 infection is underestimated in passive case- and testing-based surveillance (ie, because passive surveillance captures only those individuals who are aware of their infection and test with a provider). For example, during a 10-week period between January 1 and March 16, 2022, in a survey sample of 1,030 adults, we estimated that 27.4% (95% CI, 22.8-32.0) or 1.8 million adults (95% CI, 1.6-2.1) in New York City adults were likely infected with SARS-CoV-2 during the Omicron BA.1 surge, compared with the 552,084 cases of all ages that were reported to New York City Department of Health and Mental Hygiene during the same time period.^7^ The extent of underreporting during the Omicron BA.1 wave likely varied by time period and could be influenced by decreases in testing and the widespread uptake of at-home tests.^17^

Surveys can also estimate prevalence by sociodemographic and epidemiologic risk factors, as well as household characteristics, which can help assess epidemic trends with policy implications. This is particularly important for schools because they have increasingly underreported cases due to the expansion of at-home testing.^43^ In our survey, we found a substantially higher prevalence of SARS-CoV-2 among adults in households with children (31.5%; 95% CI, 12.9 to 43.0) than in households without children (17.8%; 95% CI, 13.6 to 22.8); this finding was robust even after controlling for household size.

Surveys can also be a powerful tool for estimating the true burden of infection at more detailed geographic levels, such as neighborhoods or counties. This is achieved through probability-based sampling and the use of case adjustment factors in combination with case- and testing-based surveillance.^44^

### Assessing and Accounting for Uptake of At-Home Testing

Rapid at-home antigen testing for SARS-CoV-2 has been implemented as a key component of the public health response to the pandemic as an individual-based testing strategy that informs behaviors to mitigate the risk of onward transmission of infection (ie, timely isolation and masking).^45,46^ However, there have been limited attempts to systematically gather and leverage at-home testing data to inform situational awareness of the levels of community spread. Currently, no mechanisms are in place that allow for the capture of positive at-home tests for surveillance purposes, and even if such mechanisms existed, reporting by testers would likely be inconsistent and unreliable for generating accurate estimates of prevalence.

Population-based surveys can gather information on the at-home use of rapid antigen tests by ascertaining information directly from the target population. Specifically, surveys can estimate the frequency of recent at-home SARS-CoV-2 test uptake and the proportion of those tests with a recent positive result that were not followed by a confirmatory test with a provider. In 2 different surveys in New York City that measured at-home testing—including probable cases and positive cases—the prevalence of SARS-CoV-2 substantially increased compared with prevalence estimates based solely on reported health and laboratory provider tests.^7,8^ Among New York City residents who tested for SARS-CoV-2, at-home test use among adults almost doubled during a 10-week period of the BA.1 surge and a 2-week period of the BA.2 surge. These findings show that a substantial proportion of the population is using at-home tests without receiving a confirmatory test.^47^

### Estimating Prevalence of SARS-CoV-2 in Medically Vulnerable Populations

In the past, CDC used commercial laboratory seroprevalence surveys to monitor the prevalence of infection-induced antibodies to SARS-CoV-2.^11^ In 2022, they began monitoring the prevalence of both vaccine- and infection-induced antibodies in blood donors.^11^ However, these indicators were not linked with cases or deaths. Apart from sentinel surveillance or special studies,^48^ there is a lack of timely, routinely collected information that incorporates medical vulnerability to severe COVID-19, history of previous SARS-CoV-2 infection, and vaccination. Such information provides context and helps determine the likelihood and extent to which surges of emerging subvariants will drive surges in severe COVID-19 outcomes, such as hospitalization, need for intensive care, and death.^49^ Conducting surveys can rapidly and efficiently ascertain when individuals receive vaccinations and boosters. In addition, having an accurate prior infection history can inform policy decisions and community mobilization efforts to encourage widespread and targeted vaccination and booster campaigns.

Using medical vulnerability to severe COVID-19 outcomes, as defined using CDC criteria,^50^ we estimated that during the BA.2/BA.2.12.1 surge in New York City,^8^ SARS-CoV-2 prevalence among medically vulnerable adults was 2.5 times higher than other adults. However, during the BA.2/BA.2.12.1 surge, we also found that 50.6% of New York City adults had hybrid protection conferred from both vaccination and prior infection, compared with 30.9% who had vaccine-induced immunity only, 11.5% with infected-induced immunity only, and 7.0% with no immunity.^8^ Our findings suggest that a smaller proportion of the population may have been vulnerable to severe outcomes during the BA.2/BA.2.12.1 surge compared with the BA.1 surge.

### Preventing and Addressing COVID-19-Related Health Inequities

It is crucial to monitor, classify, and disseminate information about the transmission of SARS-CoV-2 broken down by various subgroups based on race/ethnicity, socioeconomic status, medical vulnerability, and geographic location. This approach is essential for preventing and addressing health inequities that have been exacerbated by the COVID-19 pandemic. The CDC COVID-19 Response Health Equity Strategy is guided by data-driven approaches^51^; however, using nonrepresentative data to evaluate inequities in the population is likely to misrepresent the true population differences in the burden of infection. This is especially true given that metrics derived exclusively from the utilization of the healthcare system have missing or incomplete data on race/ethnicity, comorbidities, and important social determinants of health.^52^

During the BA.4/BA.5 surge, we found that the prevalence of SARS-CoV-2 among adults in the United States was higher among Hispanic adults and adults with lower education and income.^10^ Through surveys, it is possible to identify the populations that currently have the highest burden of infection, as well as those who are likely to experience a higher, disproportionate burden of infection and severe disease in the future (eg, undervaccinated groups and those with more comorbidities). Surveys can also help assess the effectiveness of targeted COVID-19 mitigation efforts as well as identify gaps in surveillance due to differential incompleteness of data by race, ethnicity, and other social determinants.^53^

### Capturing Pandemic Evolution: Uptake of Antiviral and Prevalence of Long COVID

Surveys can be used to capture key aspects of the public health response that are evolving, such as knowledge of and access to oral antivirals like nirmatrelvir/ritonavir (Paxlovid) and morbidity such as long COVID. For example, in a survey conducted between April 23 and May 8, 2022, we estimated that 15% (95% CI, 7.1 to 23.1) of adults in New York City with a recent SARS-CoV-2 infection reported receiving nirmatrelvir/ritonavir. Uptake varied substantially by sociodemographic groups and was higher among, for example, those with any medical vulnerability and those with health insurance. Analyzing the uptake of nirmatrelvir/ritonavir based on sociodemographic factors and medical vulnerability can reveal important inequities in antiviral access across various social determinants of health.^54^

Population-representative surveys can supplement ongoing national efforts to monitor long COVID, especially by integrating other key information on medically and socially vulnerable groups.^55^ In a cross-sectional survey of US adults conducted between June 30 and July 2, 2022, revealed that an estimated 21.5% (95% CI, 18.2 to 24.7) of people with a history of SARS-CoV-2 infection reported long COVID symptoms that varied substantially across sociodemographic characteristics.^10^ Long COVID was found to be more prevalent among respondents who were female, non-Hispanic Black, unemployed, not boosted, unvaccinated, and those who reported having comorbidities, which aligns with findings from other studies.^56^ To our knowledge, the US Census Household Pulse Survey is the only US survey that monitors long COVID on a national scale,^57^ although the survey does not integrate data on comorbidities and other related risk factors and is not representative.^58,59^

## Conclusion

During prolonged and evolving health threats of public health significance, such as the SARS-CoV-2 pandemic, traditional approaches to public health monitoring of SARS-CoV-2 have proven insufficient in the use of key health indicators that are necessary for an effective public health response. Probability-based surveys can efficiently and rapidly boost surveillance to capture key information and metrics in alignment with the dynamic public health response to the pandemic. They also have the potential to enhance surveillance in low- and middle-income countries.^60,61^

Well-conducted cross-sectional surveys for rapid monitoring of SARS-CoV-2 infection do not need to be large-scale to be useful. A high-quality random sample can yield information on a large swath of the population.^14^ Furthermore, population dynamics that influence transmission are often specific to local areas,^62^ which highlights the important role of subnational surveys to inform local public health decisions. It is important to note that although probability-based surveys of the general population might have low participation rates,^63^ knowing the sampling frame allows for easier correction of ascertainment bias compared with other surveillance approaches that rely on convenience samples (eg, internet-based or participatory surveillance). Population-based surveys are subject to selection bias that could undermine inferences related to prevalence in the population. Bias may arise at the survey sampling stage, such as when individuals invited to the survey differ from the general population, or at the participation stage when respondents differ from nonrespondents on observable or unobservable characteristics. Survey weighting methods (eg, standardization, inverse-probability weighting) can adjust for sampling and participation bias on observable respondent characteristics associated with infection.^64^ For participation bias that occurs when the likelihood of infection is dependent on the unobserved willingness or ability to participate (eg, if those with SARS-CoV-2 infections are less likely to participate), approaches such as the use of selection models^65^ can be used to ascertain, quantify the extent of, and correct for selection bias on prevalence estimates.

Resources for deploying surveys for monitoring of health outcomes exist nationally, and at many local, state, and tribal health departments. Existing surveys can be adapted to integrate COVID-19-related indicators.^66^ However, population-based surveys as a surveillance tool are best used for decisionmaking during pandemics when they are proactively deployed, and when findings are disseminated in a timely fashion to meet strategic information needs specific to infectious disease threats (ie, during surges of emerging variants). In light of these needs, pandemic preparedness requires sustained, long-term technical and financial investments, which would strengthen infrastructure and enable the release of additional resources for rapid deployment of surveys during epidemics. Such investments would strengthen health security systems both during a pandemic and during other events of public health significance.^67^

Public health goals to reduce COVID-19 morbidity and mortality have not changed. However, the COVID-19 pandemic's landscape and available tools have evolved substantially, resulting in the creation of new health intelligence needs that remain largely unmet. While there is no single approach that can meet all public health goals in responding to the pandemic, “boosting” surveillance through feasible and efficient survey-based approaches to generate timely and accurate metrics for public health decisionmaking is crucial. This approach will better serve the public during the current pandemic and future infectious disease threats.
